# Extensive arterial and venous thrombo-embolism with chemotherapy for testicular cancer: a case report

**DOI:** 10.1186/1757-1626-2-9082

**Published:** 2009-11-24

**Authors:** Ramesh Batra, Jonathan N Davies, Duncan Wheatley

**Affiliations:** 1 Surgical Directorate, Royal Cornwall Hospitals NHS Trust, Truro, UK; 2 Department of Oncology, Royal Cornwall Hospitals NHS Trust, Truro, UK

## Abstract

Germ cell tumours tend to affect young adults and with advanced treatments achieve more than 90% cure rates. Over the years cisplatin has significantly improved the relapse free survival in these patients, hence forming an essential component of chemotherapy regimes. But, the thrombo-embolic complications suffered with cisplatin significantly affect the quality of life in these young patients.

We describe a young adult who suffered a potentially fatal cerebral and pulmonary vascular insult on completing first cycle of cisplatin-based chemotherapy for a non-seminomatous germ cell tumour. Venous and arterial thrombo-embolism was the mechanism of injury and was promptly managed surgically and medically including neuro-rehabilitation.

## Introduction

Thrombo-embolism is a known vascular toxicity associated with germ cell tumours, amongst which VTE (Venous Thrombo-embolism) continues to share the highest proportion. Amongst thrombotic complications, arterial thrombosis is a rare complication of chemotherapy for testicular tumours and especially in those lacking any risk factors. Herein we describe an unusual and unfortunate case of NSGCT (Nonseminomatous germ cell tumour) who suffered an extensive internal carotid artery thrombosis and saddle pulmonary embolism after BEP (Bleomycin, Etoposide, Cisplatin) regime of chemotherapy.

## Case

**Figure 1:**
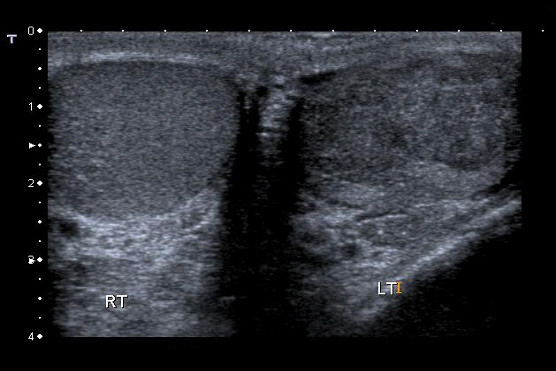
**Ultrasonograph of Testis showing the seminomatous tumour of left testis**.

**Figure 2:**
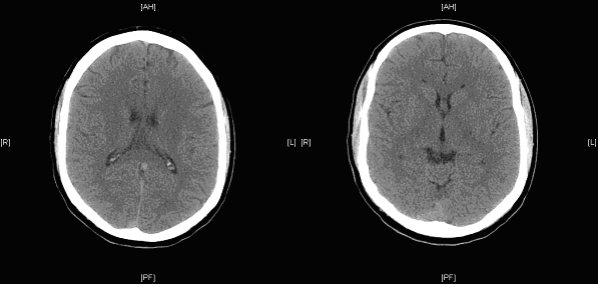
**Admission CT scan of Brain showing no evidence of Ischaemia or Intracranial bleed**.

**Figure 3:**
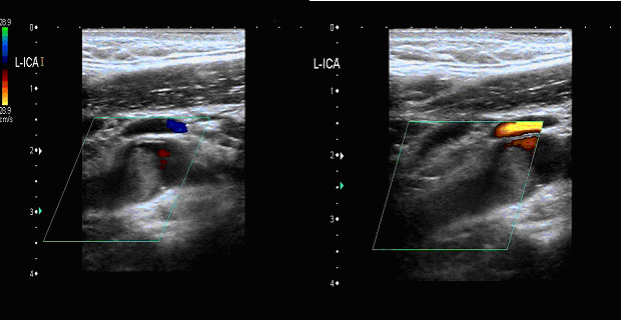
**Diagnostic Colour Doppler Ultrasonograph of Left Carotid system showing the thrombus in Left Internal Carotid Artery**.

**Figure 4:**
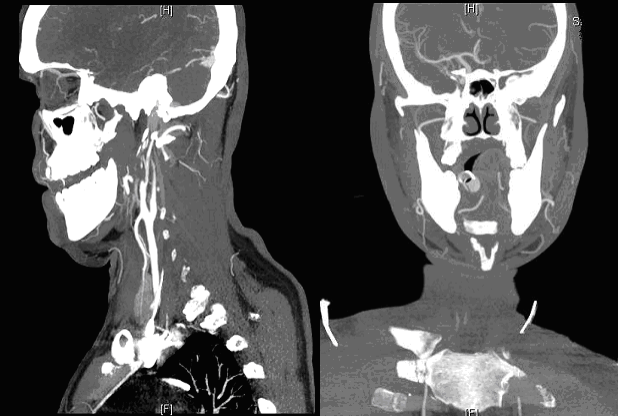
**Post-operative CT- Angiogram showing compromised Carotid and Cerebral circulation**.

**Figure 5:**
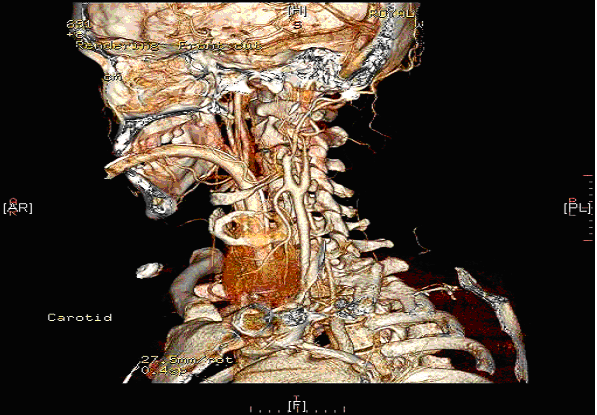
**Post-operative Remodeled CT- Angiogram showing compromised Carotid and Cerebral circulation**.

**Figure 6:**
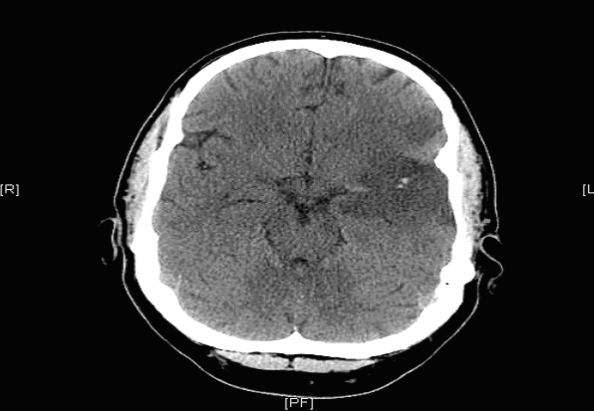
**Post operative CT scan of brain showing ischaemic changes in Left Middle Cerebral artery territory**.

A 36 year old previously fit and healthy non-smoker Caucasian male underwent left orchidectomy for a testicular tumour (Fig. 1) with AFP (alpha fetoprotein) levels of 9.7 IU/ml (reference range [RR] <10 IU/ml) and BHCG (beta human chorionic gonadotrophin) levels of <1.0 IU/L (RR < 5.0 IU/L), which turned out to be a T2N0M0 stage 1 embryonal carcinoma with vascular invasion but no local or distant metatstasis on follow-up CT (computed tomography) scan. He was kept under monthly surveillance with tumour markers, chest x-rays, and 3 monthly CT scans; wherein a scheduled CT scan revealed an ipsilateral right-sided common iliac lymph node of 15 mm and 5 mm pulmonary nodule consistent with both local and distant metastasis. He was therefore started on BEP regime of chemotherapy. However on the day of completion of first cycle of BEP regime he was re-admitted to the emergency department with complaints of sudden onset right-sided weakness and agitation. Clinical assessment revealed isolated neurological insult viz. motor power being 1/5 on the right arm and right leg with a constricted left pupil reactive to light and a GCS (Glasgow coma score) of 14 i.e. E4 V4 M6. Initial blood tests depicted normal haematology and biochemistry results with a normal magnesium of 0.89 mmol/L(RR = 0.7-1.0 mmol/L) including a normal CT brain (Fig. 2). An echocardiogram demonstrated no thrombus in heart, no atrial/ventricular septal defects, no patent foramen of ovale and no valvular vegetations. He was therefore treated for a suspicion of encephalitis secondary to chemotherapy induced immunosuppression, until a colour duplex ultrasonography of carotids (Fig. 3) revealed a dense plug of thrombus in left common carotid artery completely occluding the flow to external and internal carotid arteries. An emergency carotid thromboembolectomy was performed and the post-operative CT-angiogram of the carotids (Fig. 4) and brain (Fig. 5) revealed a further distal occlusion of the left carotid system with no flow and an established non-haemorrhagic infarct of left middle cerebral artery territory (Fig. 6). The patient was anticoagulated with heparin for 3 days and later changed to aspirin. The patient by then was densely hemiplegic on the right side with expressive and receptive aphasia and right facial nerve palsy with a non-reactive left pupil. Following which he underwent extensive neuro-rehabilitation with slow but consistent improvement in his neurological status.

The overall recovery suffered another insult by an extensive saddle pulmonary embolus for which he was anticoagulated again, only this time with warfarin. The patient's neurological state showed signs of improvement in terms of speech, motor power and sensations on right upper (power 3/5) and lower limbs (power 5/5). But two months later he demonstrated seizure activity which on MRI (magnetic resonance imaging) scan confirmed haemorrhagic transformation in the large maturing previous infarct in left middle cerebral artery territory, which was effectively controlled by anti-epileptics. He later completed his second cycle of BEP regime and a surveillance CT scan showed shrinkage of common iliac lymph node to only 7 mm and the pulmonary nodule of 4 mm. Tumour markers were normal and the plan was to continue with scheduled three monthly CT scans.

## Discussion

Cisplatin based chemotherapy as in BEP regime is considered to be highly effective in GCT, but its associated vascular complications include not only venous but also arterial thrombosis and/or hemorrhages [1-6] with the frequency of cerebrovascuar complications being less than 1 in 2000 treated patients [1]. The first reported case of cerebrovascular event following cisplatin based chemotherapy dates back to 1983 [7]. Association of cisplatin with thrombogenic events relates to the vasospasm caused by various metabolic and hormonal abnormalities. Increase in Von-willebrand factor antigen, endothelial dysfunction, alterations of clotting cascade, thromboxane-prostacyclin homeostatic disturbances, and stimulation of fibroblasts [1,7-9] add to the propensity of thrombotic events with cisplatin. Cisplatin is also known to cause autonomic dysfunction [10] and hypomagnesemia, both of which could potentiate the arterial spasm, as magnesium seems to have an important role in maintaining the tone of the muscular layer of vessels [11].

Our case suffered thrombosis of the carotid system on the day of finishing his first cycle of BEP chemotherapy, although the diagnosis of ischaemic stroke was delayed by a few hours due to the normal appearance of the immediate CT scan and hence, thought was directed towards a possibility of encephalitis secondary to chemotherapy induced immunosuppression. However the co-relation of history and clinical findings led to the early discovery of thrombosis of the carotid system on further investigations. It is difficult to demonstrate that early institution of active anticoagulation and emergency thrombo-embolectomy dictated his good neurologic recovery. However emergency surgery removed the thrombus from the left carotid system to a fairly good extent and the immediate active anticoagulation played a significant role to maintain the patency of blood-flow to the cerebral circulation.

Pretnar et al [12] described a case with similarities of ischemia in middle cerebral artery territory secondary to ICA (internal carotid artery) thrombosis in a post BEP chemotherapy patient for testicular seminoma. Our patient differed not only in suffering another fatal insult i.e. saddle pulmonary embolus, but also that the benefits of anticoagulation and surgery were well observed in his recovery.

A retrospective analysis [13] of patients with disseminated germ cell tumours treated with cisplatin-based chemotherapy revealed that probability of relapse-free survival for complete responders was 83.5%. Functional status of survivors was extremely promising with 95% returning to their pre-therapy status and 88% fully employed.

It has been demonstrated that liver metastasis, low magnesium, high dose of corticosteroids pose an additional risk of thrombo-embolic complications in germ cell tumour patients receiving chemotherapy [13]. Our patient was a nonsmoker and had none of the aforementioned risk factors for thromboembolism.

## Conclusion

Our case was unique and unfortunate in suffering from both an extensive arterial thrombosis and venous embolism a few days apart with no risk factors and a negative family history and a negative cranial CT on admission.

Since the introduction of cisplatin based chemotherapy in 1970s, patients have benefited from longer disease free survival and better cure rates for any adult malignancy [14]. The 5-year progression-free and overall survival rates for NSGCTs have reached highs of 96% and 94% respectively [15]. Our patient though unfortunate to suffer from the potentially fatal complications of cisplatin based chemotherapy achieved its associated benefits.

With our experience from this particular case we cannot emphasis enough the importance of history and clinical findings and their mutual co-relation to dictate the diagnosis with the subsequent aide of most appropriate set of investigations. Given the incidence of germ cell tumours in young patients and the high cure rates, patients stay disease free for a long time. Therefore prevention and early recognition of adverse effects of treatment is of paramount importance as it dictates the quality of life of these young patients.

## Consent

Written consent was obtained from the patient's next of kin for publication of this case report and accompanying images.

Full anonymity of our case is maintained in the case report and accompanying images.

## Competing interests

The authors declare that they have no competing interests.

## Authors' contributions

JND was the operating vascular surgeon for the patient.

RB was the vascular surgical trainee involved in the clinical management of patient, collecting the information and editing it for submission.

DC was the oncologist involved in the care of our patient.
